# Does color matter? Molecular and ecological divergence in four sympatric color morphs of a coral reef fish

**DOI:** 10.1002/ece3.6566

**Published:** 2020-09-03

**Authors:** Michelle R. Gaither, Darren J. Coker, Samuel Greaves, Fatih Sarigol, Samuel D. Payet, Veronica Chaidez, Tane H. Sinclair‐Taylor, Joseph D. DiBattista, Michael L. Berumen

**Affiliations:** ^1^ Genomics and Bioinformatics Cluster Department of Biology University of Central Florida Orlando FL USA; ^2^ Division of Biological and Environmental Science and Engineering Red Sea Research Center King Abdullah University of Science and Technology Thuwal Saudi Arabia; ^3^ Faculty of Biology Ludwig‐Maximilians‐Universität München Planegg‐Martinsried Germany; ^4^ Australian Institute of Marine Science Townsville QLD Australia; ^5^ Trace and Environmental DNA Laboratory School of Molecular and Life Sciences Curtin University Perth WA Australia; ^6^ Australian Museum Research Institute Australian Museum Sydney NSW Australia

**Keywords:** behavior, color polymorphism, life history, outlier loci, population structure, RADSeq

## Abstract

Non‐sex‐linked color polymorphism is common in animals and can be maintained in populations via balancing selection or, when under diversifying selection, can promote divergence. Despite their potential importance in ecological interactions and the evolution of biodiversity, their function and the mechanisms by which these polymorphisms are maintained are still poorly understood. Here, we combine field observations with life history and molecular data to compare four sympatric color morphs of the coral reef fish *Paracirrhites forsteri* (family Cirrhitidae) in the central Red Sea. Our findings verify that the color morphs are not sex‐limited, inhabit the same reefs, and do not show clear signs of avoidance or aggression among them. A barcoding approach based on 1,276 bp of mitochondrial DNA could not differentiate the color morphs. However, when 36,769 SNPs were considered, we found low but significant population structure. Focusing on 1,121 *F*_ST_ outliers, we recovered distinct population clusters that corresponded to shifts in allele frequencies with each color morph harboring unique alleles. Genetic divergence at these outlier loci is accompanied by differences in growth and marginal variation in microhabitat preference. Together, life history and molecular analysis suggest subtle divergence between the color morphs in this population, the causes for which remain elusive.

## Graphical abstract

Despite their potential importance in ecological interactions and the evolution of biodiversity, non‐sex‐linked color polymorphism is poorly understood. Here, we investigated four sympatric color morphs of the coral reef fish *Paracirrhites forsteri* (family Cirrhitidae) in the central Red Sea and show that the color morphs inhabit the same reefs. While we could not differentiate between them using mtDNA, when we considered 36,769 SNPs, we found low but significant population structure that corresponded to color morphology with accompanying differences in growth and marginal variation in microhabitat preference.

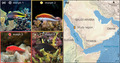

## 1 INTRODUCTION

Color polymorphism is common among animals and serves a number of critical functions in communication, crypsis, mate signaling, and mimicry (McLean & Stuart‐Fox, 2014; Nosil & Crespi, 2006; Roulin, 2004; White & Kemp, 2016). Theoretical models predict that balancing selection can maintain color polymorphisms in a population under a number of scenarios (Gray & McKinnon, 2006). For instance, distinct color morphs may be maintained if they differ in their susceptibility to predation in conflicting visual environments (Farallo & Forstner, 2012; Hurtado‐Gonzales, Loew, & Uy, 2014) or if the fitness of a morph changes relative to the abundance of another morph (frequency‐dependent selection; Bond, 2007; Le Rouzic, Hansen, Gosden, & Svensson, 2015; Olendorf et al., 2006; Seehausen & Schluter, 2004). In some freshwater and terrestrial animals, differences in predation success between habitats have been shown to enable the maintenance of color morphs (Kohda & Hori, 1983; Nshombo, 1994), as has defensive mimicry of aggressive or toxic species (Long, Hahn, & Shapiro, 2014) or aggressive mimicry of nonpredatory species (Hori & Watanabe, 2000). Color polymorphism has been shown to influence mate choice and social interactions in a diversity of animals (Coyne & Orr, 2004), indicating that individuals can assess and use color morphology in social decision making (i.e., Endler, 2011; Hurtado‐Gonzales et al., 2014; Roulin, 2004). The mechanisms by which these polymorphisms arise and are maintained within populations are still poorly understood, but recent studies show evidence of extensive variation at the chromosomal level among color morphs of stick insects (Lindtke et al., 2017) and moths (van't Hof et al., 2016).

Coral reef fishes often exhibit bright and conspicuous colors. Although little more than color pattern distinguishes some species, coloration is not always a diagnostic characteristic (DiBattista, Gaither, Hobbs, Rocha, & Bowen, 2016, 2017; DiBattista, Gaither, Hobbs, Saenz‐Agudelo, et al., 2017; Gaither et al., 2014; Schultz, Pyle, DeMartini, & Bowen, 2007; Sorenson, Allen, Erdmann, Dai, & Liu, 2014; Taylor & Hellberg, 2005). In some cases, color polymorphism has been observed within and among populations of reef fishes and has been linked to sex, life history stage, or behavior (Thresher, 1984; Thresher & Moyer, 1983). Of particular interest is color polymorphism in fish that is not linked to sex or ontogeny, but instead may represent intraspecific variation or unrecognized evolutionary partitions (Violi, Gaither, Burns, Hoelzel, & Neat, 2018; Whitney, Bowen, & Karl, 2018). Where color morphs are geographically isolated, molecular evidence often indicates recent divergence that may not relate to ecologically or behaviorally driven evolution (Drew, Allen, & Erdmann, 2010; Drew, Allen, Kaufman, & Barber, 2008; Taylor & Hellberg, 2003). When found in sympatry, color morphology can fail to translate into genetic partitions (Lin, Sánchez‐Ortiz, & Hastings, 2009; Messmer, van Herwerden, Munday, & Jones, 2005), implicating a mechanism other than geographic isolation in their maintenance (Messmer et al., 2005; Munday, Eyre, & Jones, 2003).

Hawkfishes in the family Cirrhitidae are small reef predators that often perch on or shelter within scleractinian corals. There are 34 recognized species within the family (Gaither & Randall, 2012; Randall, 2001), four of which occur in the Red Sea, including one endemic species recently resurrected from synonymy (DiBattista, Roberts, et al., 2016; Gaither & Randall, 2013). Stable and sympatric color morphs have been documented in three of the six species in the genus *Paracirrhites* (*P. forsteri*, *P. arcatus*, and *P. hemistictus*) (Randall, 2005; Whitney, Donahue, & Karl, 2018). The two non‐sex‐linked color morphs of *P. arcatus* show a strong correlation between phenotype and environment on Hawaiian reefs (Whitney, Donahue, et al., 2018), as well as significant divergence at microsatellite loci and a gene associated with coloration (Whitney, Bowen, et al., 2018). Taken together, these data support the possibility of at least partial assortative mating among the two color morphs of *P. arcatus*. *Paracirrhites forsteri* (Bloch & Schneider, 1801) is unique among hawkfishes in that it displays at least four color morphs (vs. two morphs in *P. arcatus* and *P. hemistictus*) whose relationship to sex and ontogeny remains unresolved (Coker, Chaidez, & Berumen, 2017; Donaldson, 1990; Myers, 1989; Randall, 1963, 2005). The color morphs vary in abundance throughout the Indo‐West Pacific, in some cases exhibit geographic variation, are indistinguishable based on meristic characters (Myers, 1989; Randall, 2005), and sometimes display intermediate coloration.

Here, we combine ecological field observations with life history and molecular data to determine (a) whether color morphology in *P. forsteri* is linked to sex or ontogeny, (b) whether life history traits such as growth rates differ among the four morphs, and (c) whether color morphs can be distinguished using mitochondrial DNA (mtDNA) and genome‐wide single nucleotide polymorphisms (SNPs). By sampling the four morphs from the same reefs in the central Red Sea, we assess whether these sympatric color morphs demonstrate genetic divergence without the confounding effects of geography.

## 2 MATERIALS AND METHODS

### 2.1 Life history metrics and in situ behavioral observations

**FIGURE 1:**
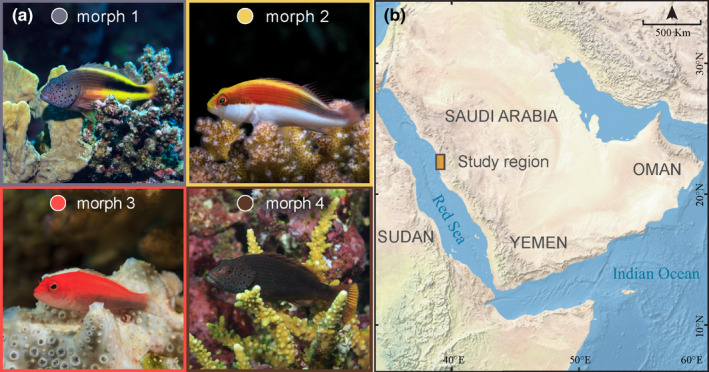
Color morphs and sampling location. (a) The four color morphs of *Paracirrhites forsteri* and (b) study region within the central Red Sea

Specimens of all four color morphs of *P. forsteri* were collected from midshelf (~14 km from shore) and offshore reefs (~56 km from shore) in the central Red Sea near Thuwal in Saudi Arabia while scuba diving or snorkeling (Figure 1; Table S1). A total of 193 individuals were used to investigate life history characteristics among the four morphs (Table S1). Fish were sexed using visual assessments of the gonads following Mackie, Lewis, Gaughan, and Newman (2005). Specifically, gonads were dissected and examined for the presence of vitellogenic oocytes (females) and milt sperm tissue (males). Of the 193 individuals collected, 121 or 63% could be confidently assigned a sex, however within Morph 3, only 33% could be assigned a sex. Across all four morphs, 166 otoliths were processed of which 147 could be used to resolve age estimates (following Chan & Sadovy, 2002). Otoliths were processed by conducting two blind reads on each otolith in order to quantify the precision of counts (Taylor & McIlwain, 2010). Nine otoliths could not be consistently read (defined as a difference of >2 years between reads) and were excluded from analyses. Growth characteristics of each morph were modeled based on total length (TL, mm) and age (years) using the von Bertalanffy growth function (VBGF), which is described by the equation: *L_t_* = *L*_∞_[1 – *e* − *k*(*t* − *t*_0_)], where *L_t_* = total length of fish of age *t*(years); *L_∞_* = asymptotic mean total length; *k* describes the curvature of growth toward *L*_∞_; *t* = age of the fish; and *t*_0_ = the hypothetical age at which the mean length is zero if it had always grown in a manner described by the VBGF. Length at age zero (*L*_0_) was fixed at 10 mm for each color morph to improve estimates and comparisons of growth parameters (Kritzer, Davies, & Mapstone, 2001). Growth rates were compared following the methods described by Rhodes, Taylor, and McIlwain (2011). We plotted ellipsoidal 95% bivariate confidence intervals around estimates of the growth coefficient (*K*) and the mean asymptotic total length (*L_∞_*) for each morph. Overlapping ellipses were considered to be statistically similar.

In situ behavioral observations were conducted on Al Fahal Reef (Thuwal) where all four color morphs are found in high densities (Coker et al., 2017). A total of 52 individuals across a range of sizes from all morphs were observed for 10 min each. During observations, we noted for each individual (a) color morph, (b) total length (TL, estimated visually), (c) distance to (to the nearest 10 cm) and the morph of the nearest conspecific, and (d) evidence of aggression among morphs in an attempt to identify dominance hierarchies and competition. Aggressive interactions were observed as an individual chasing another or forcing another individual from a perch or habitat.

### 2.2 Sanger sequencing and mtDNA analysis

Specimens used for genetic analysis are listed in Table S2. Some of these individuals were also used to calculate growth and life history parameters (see above), but there is not complete overlap of specimens. Total genomic DNA was extracted using the “HotSHOT” protocol of Meeker, Hutchinson, Ho, and Trede (2007). A 585‐bp fragment of the mitochondrial cytochrome c oxidase subunit I gene (COI) and a 691‐bp fragment of cytochrome *b* (CytB) gene were amplified using the primers FishF2 and FishR2 (Ward, Zemlak, Innes, Last, & Hebert, 2005) or Cyb9 (Song, Near, & Page, 1998) and Cyb7 (Taberlet, Meyer, & Bouvet, 1992), respectively. PCRs were carried out, and products were prepared for sequencing following DiBattista et al. (2012), with annealing temperatures set at 50°C for COI and 58°C for CytB. DNA was sequenced in both directions on an ABI 3130XL Genetic Analyzer (Applied Biosystems). The sequences were aligned, edited, trimmed, and concatenated using geneious pro v6.1.8 (Kearse et al., 2012). Individual mtDNA sequences are deposited in GenBank, and associated metadata is available at GEOME (Deck et al., 2017) and provided in Table S2.

We calculated haplotype, nucleotide diversity, and Fu's *F*_S_ using 50,000 permutations in arlequin v3.5.1.2 (Excoffier, Laval, & Schneider, 2005). An unrooted neighbor‐joining tree was constructed using 1,000 bootstraps in mega v5.05 (Tamura, Dudley, Nei, & Kumar, 2007). The best‐fit general substitution model was selected using the Bayesian information criterion employed in mega (Tamura & Nei, 1993) and implemented with all codon positions selected. Bootstrap support values were calculated using 1,000 replicates.

### 2.3 RADSeq library preparation, sequencing, and analysis

We prepared RADSeq libraries using the double‐digest protocol of Peterson, Weber, Kay, Fisher, and Hoekstra (2012) following Gaither et al. (2015). Loci were assembled using the denovo_map.pl pipeline of stacks v2.1 and its component programs (Catchen, Amores, Hohenlohe, Cresko, & Postlethwait, 2011). Loci were generated by the merging of three or more “stacks,” allowing two mismatches between loci (‐n) when building the catalog and three mismatches when processing a single individual (‐M). Stacks in which the numbers of reads were more than two standard deviations above the mean were assumed to be repetitive elements and removed from the dataset. Due to potential PCR error, we removed all singleton loci and loci in which rare alleles occurred in only a single individual from the dataset. Using the “populations” component in stacks, we further filtered the dataset, retaining only those loci that amplified in ≥80% of individuals per population and those that were found in all four color morphs, which resulted in 36,812 loci. Output files were created by implementing the “write_single_snp” option. We tested loci for significant deviation from Hardy–Weinberg equilibrium using arlequin v3.5 (Excoffier, Laval, & Schneider, 2010). After correcting for multiple comparisons (Benjamini & Yekutieli, 2001; Narum, 2006), 43 loci were found to deviate from HWE expectations in two or more populations, which were then removed leaving a final dataset of 36,769 SNPs.

### 2.4 Identifying outlier loci and genetic structure

It is important to note that while outlier methods are often used to identify loci under selection, this was not our goal. Instead, our aim was to determine whether color morphs could be distinguished by loci with elevated *F*_ST_ values. With this goal in mind, we used two methods to identify *F*_ST_ outliers. First, we ran the Fdist approach (Beaumont & Nichols, 1996) as implemented in lositan (Antao, Lopes, Lopes, Beja‐Pereira, & Luikart, 2008). We ran 100,000 simulations under the infinite alleles model with a false discovery rate of 0.05 and the options of neutral mean *F*_ST_ and forced mean *F*_ST_. We identified only those SNPs outside the 99% confidence interval as outliers. Next, we used the *F*_ST_ values calculated by genepop v4.7 (Weir & Cockerham, 1984) and considered those loci with *F*_ST_ values greater than two standard deviations above the mean *F*_ST_ (2SD) as outliers (modified from Bernardi, Nelson, Paddack, Rulmal, & Crane, 2018).

To test for population structure, an analysis of molecular variance (AMOVA) was performed in arlequin using 50,000 permutations for all loci, the lositan outliers, and 2SD outliers. Overall *F*_ST_ was calculated for each dataset as well as for pairwise comparisons among locations. We evaluated population clustering using the discriminate analysis of principal component (DAPC) method (Jombart, Devillard, & Balloux, 2010) implemented in the R package adegenet v2.1 (Jombart, 2008; Jombart & Ahmed, 2011). We retained 29 PCs or *N*/3 (number of samples/3) PCs for each analysis and used the “assignplot” function to obtain population probability assignments for each individual. Next, we employed the Bayesian clustering algorithm of structure v2.3.2 (Hubisz, Falush, Stephens, & Pritchard, 2009; Pritchard, Stephens, & Donnelly, 2000). The simulations were run without a priori information about population assignment. The analyses were run with the admixture model and with correlated allele frequencies (Falush, Stephens, & Pritchard, 2003), with a burn‐in period of 100,000 MCMC iterations, followed by 500,000 iterations for each run. Eight replicates of each simulation from *K* = 1–4 genetic clusters were run for each dataset. The structure results were analyzed, the most likely number of genetic clusters (*K*) was determined (Evanno, Regnaut, & Goudet, 2005), and the results were visualized using the Clustering Markov Packager Across K (clumpak) online tool (Kopelman, Mayzel, Jakobsson, Rosenberg, & Mayrose, 2015).

## 3 RESULTS

### 3.1 Life history metrics and ecological interactions among color morphs

**FIGURE 2:**
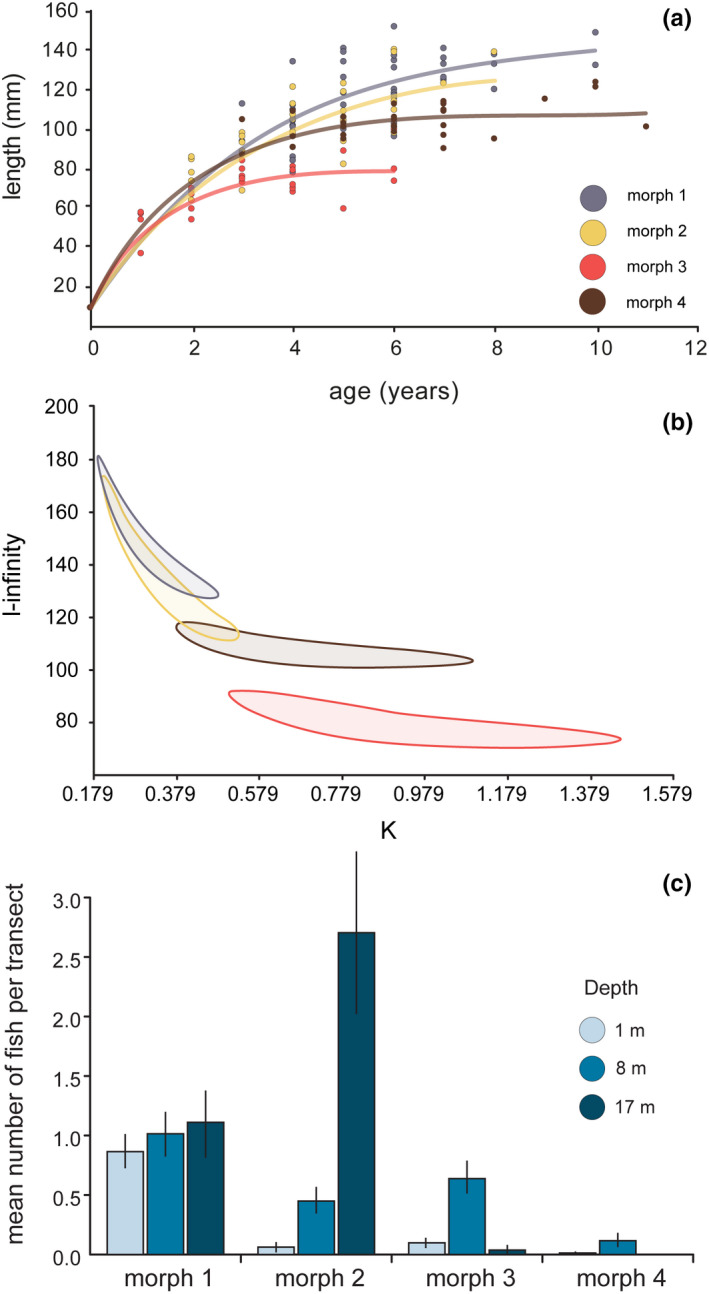
Growth data and depth distribution. (a) von Bertalanffy growth functions. (b) Comparisons of growth parameters between the four color morphs using bivariate 95% confidence ellipses surrounding estimates of *K* and *L*_∞_. (c) Distribution densities (mean ± *SE*) of the four color morphs recorded through underwater visual census across a depth gradient (data modified from Coker et al., 2017)

Examinations of gonads showed that color morphology is not sex‐linked, with males and females identified in all four morphs (Appendices A and B). The age ranges for individuals we were able to successfully evaluate were 2–6, 1–8, 1–6, and 3–11 years old for Morphs 1–4, respectively (Table S1). The smallest individuals (TL) collected were 78, 30, 21, and 78 mm, whereas maximum sizes were 152, 140, 100, and 124 mm for Morphs 1–4, respectively (Appendix B, Table S1). Overlapping 95% confidence limits surrounding estimates of growth rate (*K*) and asymptotic size (*L_∞_*) indicate that these parameters were statistically similar between Morphs 1 and 2 (Figure 2a,b; Appendix C), which were the largest and slowest growing individuals (Figure 2). Morph 4 had marginally faster growth rates than Morphs 1 and 2 and achieved a smaller asymptotic size, whereas Morph 3 had the smallest asymptotic size and demonstrated a significantly faster growth rate than the other morphs (Figure 2a,b).

Transects indicate that Morphs 1 and 2 were the most abundant and that the latter preferred deeper habitat (Figure 2c). The males of Morphs 1 and 2 tended to be larger which may reflect protogynous hermaphroditism which is thought to characterize this species (Donaldson, 1990). In situ field observations of 52 individuals for a total of 510 min revealed no clear patterns in aggression or dominance among morphs. Multiple morphs co‐occurred on exposed reef slopes (Appendix D) and were often seen in close proximity to each other (<1 m). For approximately half of the observed individuals (24 of 52), the nearest conspecific was a different morph with an average minimum distance of 1.05 m (range, 0.1–2.0 m). On nine occasions, multiple morphs were observed perched on a single coral colony. Within the study region, individuals displayed minimal levels of aggression, with only nine occurrences (chasing another individual from a perch or habitat) recorded during the total observational period. Of these nine aggressive interactions, eight occurred between different morphs. Even though *P. forsteri* were often perched on the top of exposed coral heads, we did not observe any predation attempts toward this species by larger fishes.

### 3.2 Sanger sequencing of mtDNA reveals no genetic divergence

**FIGURE 3:**
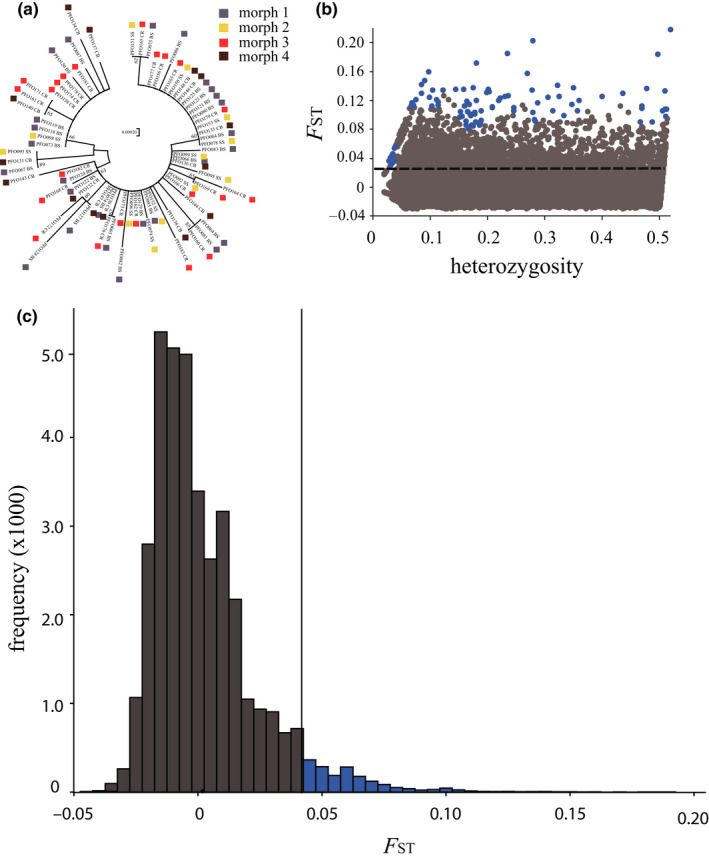
Mitochondrial sequence and SNP data. (a) Neighbor‐joining phylogenetic tree for 1,276 bp of mitochondrial data from the four color morphs. The optimal unrooted tree with the sum of branch length = 0.027 is shown, and only bootstrap values >60 are displayed. (b) Results of lositan outlier analysis of RADSeq loci. Loci in blue are statistical outliers, and dashed line indicates the lowest *F*_ST_ value for which an outlier was detected. (c) Frequency distribution of *F*_ST_ values for 36,769 SNPs. The solid black line indicates two standard deviations (2SDs) above the mean *F*_ST_ value while the blue bars show the loci that fall above this value

We amplified 1,276 bp of mtDNA (COI, 585 bp; CytB, 691 bp) in 74 individuals (Table S2). Molecular diversity indices were similar among color morphs; haplotype diversity was high and ranged from 0.86 to 0.92, whereas nucleotide diversity ranged from 0.001 to 0.002. Fu's *F*_S_ was significant and negative in all morphs indicating similar population histories of recent expansion (Appendix E). Sequence divergence across the entire dataset was low with an average divergence of 0.2% between the color morphs (interspecific divergences among species of *Paracirrhites* range from 6% to 10% by comparison) (Gaither & Randall, 2012; Ratnasingham & Hebert, 2007). The most divergent sequences were only 0.5% different. Overall *F*_ST_ was −0.023 and not significant. The unrooted neighbor‐joining tree indicated no clustering of haplotypes (Figure 3a) and supported the conclusion of no mtDNA divergence between color morphs.

### 3.3 Analyses of the nuclear SNP dataset reveal divergence in outlier loci

After filtering our high‐throughput sequencing data, we resolved 36,769 SNPs in 87 individuals across the four color morphs (Table S2; M1 = 28, M2 = 18, M3 = 27, and M4 = 14). Based on the lositan results, 1,121 loci were identified as outliers (Figure 3b) whereas 1,690 loci were determined to be two standard deviations (2SD; *F*_ST_ > 0.044) above the mean *F*_ST_ (mean *F*_ST_ = 0.002) calculated using genepop v4.7 (Weir & Cockerham, 1984) (Figure 3c), with 406 loci overlapping between the two methods. Analyses of the two outlier datasets resulted in similar patterns based on DAPC, but structure runs for the 2SD outliers failed to consistently resolve the color morphs. Here, we present only the lositan dataset in the main text while results for the complete dataset and the 2SD dataset are presented in Appendix F.

**FIGURE 4:**
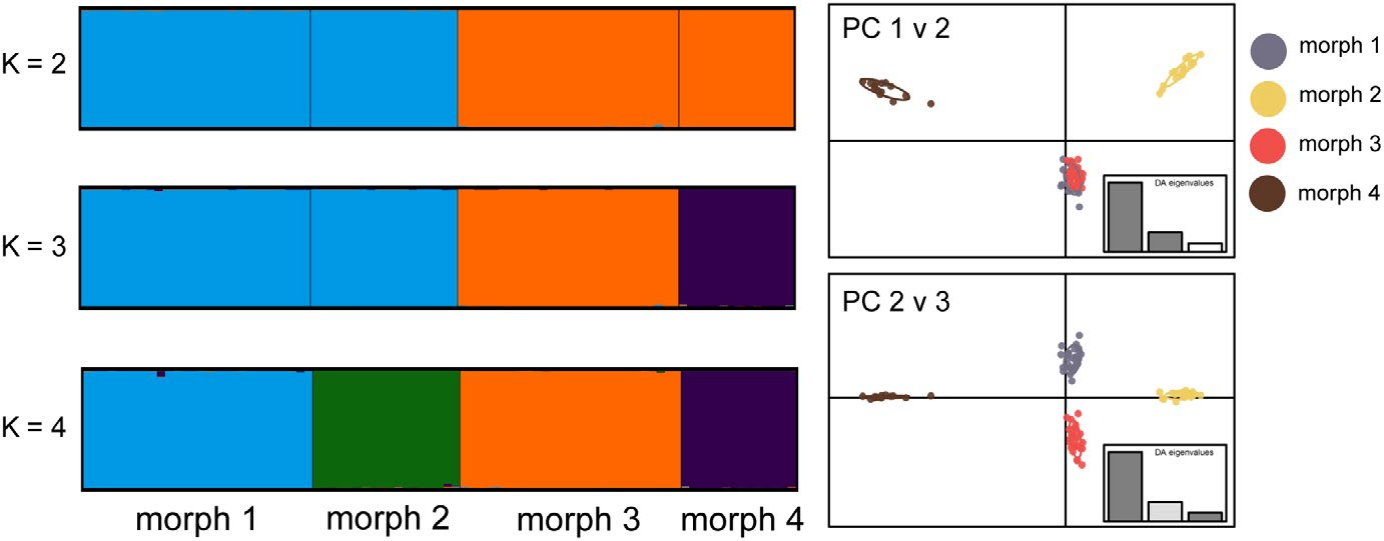
Results of Bayesian cluster analyses and discriminate analysis of principal components (DAPC). structure and DAPC plots for the 1,121 lositan outlier loci (above 99% CI). The most likely *K* identified by structure harvester was *K* = 4.

When analyzing all 36,769 loci, we found low but significant population structure (*F*_ST_ = 0.002; *p* < .001). Pairwise comparisons between color morphs showed low but significant population structure between each morph (*F*_ST_ range = 0.001–0.003; Table 1). However, the analyses using structure and DAPC could not delineate any of the color morphs (Appendix F). When only the lositan outliers were considered, overall *F*_ST_ (*F*_ST_ = 0.064; *p* < .001) and each pairwise *F*_ST_ value were significant (*F*_ST_ range = 0.041–0.103; Table 1). DAPC analysis of these outliers resulted in a distinct pattern of clustering that distinguished each of the color morphs (Figure 4), a pattern that was corroborated by the results from structure (most likely *K* identified by structure harvester was *K* = 4; Figure 4).

**TABLE 1:** Pairwise *F*‐statistics for the four color morphs of *Paracirrhites forsteri*. *F*_ST_ values calculated in arlequin are shown below the diagonal, while associated (uncorrected) *p*‐values are above diagonal. Values for all loci, as well as only the lositan outliers, are shown

All loci				
	M 1	M 2	M 3	M 4
**M 1**	–	0.007	0.000	0.003
**M 2**	0.001	–	0.000	0.001
**M 3**	0.002	0.003	–	0.001
**M 4**	0.001	0.003	0.003	–

Outlier loci				
	M 1	M 2	M 3	M 4
**M 1**	–	0.000	0.000	0.000
**M 2**	0.061	–	0.000	0.000
**M 3**	0.041	0.063	–	0.000
**M 4**	0.072	0.103	0.074	–

## 4 DISCUSSION

Non‐sex‐linked color polymorphism is a common phenomenon among the brightly colored coral reef fishes, and yet, its role in ecology and evolution is still poorly understood. In allopatry, divergent color morphology is often accompanied by genetic partitions (Drew et al., 2008, 2010; Taylor & Hellberg, 2003). However, published studies on divergent non‐sex‐linked color morphs in sympatry are rare (Whitney, Bowen, et al., 2018; Whitney, Donahue, et al., 2018). *Paracirrhites forsteri* displays sympatric color morphs that vary in appearance and abundance throughout the Indo‐West Pacific. Here, we demonstrate that color morphs on central Red Sea reefs are not sex‐linked (Appendix A), suggesting that sexual selection does not promote color polymorphism in this species. Only minor differences in microhabitat preference have been detected among the morphs on these reefs, along with differences in abundance with depth (Coker et al., 2017) (Figure 2c). Contrasting growth characteristics may reflect undetermined differences in diet or alternate life history strategies, where the slower growing morphs mature later in life but their reproductive period is extended due to greater longevity. Based on the size distribution data (Appendix B), we could not rule out that color morphology in at least one morph (Morph 3) is linked to ontogeny; however, our genetic data do not support this hypothesis (Myers, 1989). Analyzing over 36,000 SNPs, we detected low but significant population structure. When we focused our analyses on the outlier loci, we found that both *F*‐statistics and clustering analyses differentiate the four color morphs of *P. forsteri*. We did not find fixed (diagnostic) genetic differences between morphs; instead, we recorded significant shifts in allele frequencies among the outliers, with each morph harboring unique low‐frequency alleles. Together, the evidence indicates that balancing selection or possibly nonrandom mating is maintaining these color morphs. We assert that the color morphs of *P. forsteri* in the central Red Sea represent another fascinating case of stable sympatric color morphs among hawkfishes in the genus *Paracirrhites* and present this group as a model system for in‐depth study of color polymorphism in reef fishes.

### 4.1 Maintenance of permanent non‐sex‐linked color polymorphisms

Studies in terrestrial and freshwater systems have shown that color polymorphism can be maintained in a population under a number of scenarios including spatial, temporal, or frequency‐dependent variation in the relative fitness of different morphs (Futuyma, 1998; Gray & McKinnon, 2006; Rausher, 1984). The few studies that explicitly examine the adaptive advantage of color polymorphism in marine systems involve aggressive mimicry. For example, the brown dottyback (*Pseudochromis fuscus*) displays two color morphs (yellow and brown) on the Great Barrier Reef that spatially segregate and associate with similarly colored species of damselfish of the genus *Pomacentrus* (Messmer et al., 2005; Munday et al., 2003). However, in this case polychromatism is not permanent but instead individuals change color if the model species is altered. Studies show that in this system, prey capture success increases when the mimic/model is correctly matched (Cortesi et al., 2015). Probably, the best‐studied example of aggressive mimicry in a coral reef fish is among the eleven color morphs of Caribbean hamlets of the genus *Hypoplectrus*, which are known to exhibit assortative mating and in which some color morphs mimic nonpredatory fishes (Picq, McMillan, & Puebla, 2016; Puebla, Bermingham, Guichard, & Whiteman, 2007; Puebla, Bermingham, & McMillan, 2014).

There is no evidence of mimicry in *P. forsteri*, and these lie‐in‐wait predators do not routinely associate with other species, but instead, color pattern is thought to afford them some degree of camouflage against reef habitats. Indeed, evidence of increased susceptibility to predation has been documented in coral‐dwelling damselfishes after a bleaching event (Coker, Pratchett, & Munday, 2009). Moreover, studies on freshwater guppies and terrestrial snakes indicate that distinct color morphs may differ in their susceptibility to predation in contrasting visual environments (Farallo & Forstner, 2012; Hurtado‐Gonzales et al., 2014). Conversely, crypsis has been linked to differential prey capture success among color morphs of African cichlids (Kohda & Hori, 1983; Nshombo, 1994). Whether the result is a decrease in predation risk or an increase in the success of prey capture (or both), crypsis is a common strategy among animals (Kohda & Hori, 1983) and has been posited to provide an adaptive advantage in the congeneric hawkfish *P. arcatus* (DeMartini & Donaldson, 1996; Whitney, Donahue, et al., 2018). While crypsis has not been confirmed in *P. forsteri* and no predation events were observed in this study, the darker morphs of this species (Morphs 1 and 4) have been observed to retreat to dead coral habitats when threatened, leading to the hypothesis that their dark color affords them additional camouflage when seeking refuge inside the reef structure (Coker et al., 2017). However, the lack of observed differences in habitat use among the four color morphs means that the ecological significance of color morphology in this species remains equivocal.

### 4.2 Ontogenetic shifts or genetically divergent morphs?

Individuals of some species change color patterns based on ontogeny, habitat use, deception, or communication (Ng, Geneva, Noll, & Glor, 2017). While rare, individuals with a blended color pattern have been observed in *P. forsteri* in the Red Sea (D.C. personal observation). However, there is no direct evidence that color pattern is linked to ontogeny in *P. forsteri* in the Red Sea (as had been proposed in Pacific Ocean populations; Randall, 2005, 2007). Moreover, our genetic clustering analyses indicate that there are genetic distinctions between the color morphs. While no individuals younger than 2 and 3 years of age were collected or observed in this study for Morphs 1 and 4, respectively (Appendix B), other surveys in the Red Sea have documented multiple individuals of Morph 1 at ~50 mm in length. Based on the length‐at‐age curves (Figure 2a), these individuals would be approximately one year old (D. Coker, unpublished data). However, the paucity of small individuals of some morphs raises the question of where the youngest age classes in these morphs reside. One possible explanation for the missing small size classes is that the juveniles and young fish spend most of their time hiding within the coral reef structure and therefore evade capture/observation by scuba divers. Support for this can be found from studies on Red Sea reefs using ichthyocides. In one case, at least four small‐sized individuals of *P. forsteri* (<40 mm; estimated <1 year old) were captured using this method, including an individual of Morph 1 (Coker, DiBattista, Sinclair‐Taylor, & Berumen, 2018). Furthermore, individuals of Morphs 1 and 4 may reside in the reef structure longer than the other morphs explaining their absence from our visual surveys. This scenario is, in part, supported by the finding that Morph 3 grows faster than the other morphs (Figure 2a,b) and reaches its maximum size at an earlier age (~3 years), and thus may transition to adult behavior sooner than the other morphs. Further work is needed to fully confirm this hypothesis.

## 5 Conclusion

Non‐sex‐linked color polymorphism can be maintained by balancing selection, but when under the influence of divergent selection, it can promote speciation. Unique color morphology can be used to distinguish allopatric populations diverging through neutral processes. In these cases, color polymorphism is often accompanied by concordant differences at the genomic level. However, the persistence of stable non‐sex‐linked color morphs in sympatry is not well studied in the marine environment and yet may be key to understanding how populations diverge despite gene flow. Here, we confirm that color morphology is not sex‐linked in the four sympatric color morphs of *P. forsteri* in the Red Sea. We observed color morphs occupying the same reefs, and sometimes the same coral heads, with little evidence of avoidance or aggressive behaviors. Mitochondrial DNA did not delineate the four morphs; however, when we analyzed a large SNP dataset, and in particular the outlier loci, we detected significant shifts in allele frequencies, unique low‐frequency alleles among morphs, and distinct population clusters that corresponded to color morphology. Differences in growth among the color morphs seen here and the slight variation in microhabitat preference previously reported (Coker et al., 2017) suggest that either balancing selection is maintaining color morphs in this species or they may represent signals of historical isolation.

## CONFLICT OF INTEREST

The authors declare no conflict of interest.

## AUTHOR CONTRIBUTIONS

**Michelle R. Gaither:** Conceptualization (lead); Data curation (lead); Formal analysis (lead); Funding acquisition (supporting); Investigation (lead); Methodology (lead); Project administration (lead); Writing‐original draft (lead); Writing‐review & editing (lead). **Darren J. Coker:** Conceptualization (supporting); Data curation (lead); Formal analysis (supporting); Methodology (supporting); Writing‐original draft (supporting); Writing‐review & editing (supporting). **Samuel Greaves:** Formal analysis (supporting); Methodology (supporting). **Fatih Sarigol:** Formal analysis (supporting). **Samuel Payet:** Data curation (lead); Formal analysis (lead); Methodology (lead); Writing‐original draft (supporting); Writing‐review & editing (supporting). **Veronica Chaidez:** Data curation (supporting); Methodology (supporting). **Tane H. Sinclair‐Taylor:** Formal analysis (supporting); Visualization (lead). **Joseph Dibattista:** Data curation (supporting); Funding acquisition (supporting); Writing‐original draft (supporting); Writing‐review & editing (supporting). **Michael Berumen:** Funding acquisition (lead); Project administration (supporting); Resources (lead); Writing‐original draft (supporting); Writing‐review & editing (supporting).

### OPEN RESEARCH BADGES

This article has earned an Open Data Badge for making publicly available the digitally‐shareable data necessary to reproduce the reported results. The data are available at NCBI and GEOME.

## Supporting information

Tables S1andS2

## Data Availability

mtDNA COI (accession numbers: MT611991–MT612075) and Cytb sequences (accession numbers: MT596962–MT597039), as well as RADSeq fastq files (accession numbers: SRR12037068–SRR12037154), are deposited in NCBI's GenBank and SRA, respectively (Table S2). Associated metadata is available at GEOME (Deck et al., 2017) and is provided in Table S2.

## APPENDIX A

### Sample sizes and proportions (%) of male and females for each color morph

**Table:**

	Number of individuals	Number sexed	Males	Females	% males	% females
Morph 1	61	37	23	14	62	38
Morph 2	51	38	22	16	58	42
Morph 3	45	15	6	9	40	60
Morph 4	36	31	5	26	16	84

## APPENDIX C

### Summary of growth metrics. Values were calculated by the von Bertalanffy growth function: *L_t_* = *L_∞_* [1 − e − *K* (*t* − *t*_0_)], where *L_t_* = mean total length of fish of age *t*; *L_∞_* = asymptotic mean total length; *K* = a rate constant that determines the rate at which *L_t_* approaches *L_∞_*; *t* = age of the fish; *t*_0_ = the hypothetical age at which the mean length is zero if it had always grown in a manner described by the VBGF; *r* ^2^ = the fit of the curve to the data; *t*_max_ = maximum observed age

**Table:**

	*L_∞_*	*K*	*t*_0_	*r* ^2^	*t*_max_	*N*
Morph 1	147	0.3	−0.24	.42	10	47
Morph 2	133	0.33	−0.23	.6	8	40
Morph 3	80	0.82	−0.16	.56	6	27
Morph 4	108	0.59	−0.17	.19	11	28

## APPENDIX D

### Distribution data from Red Sea reefs. Number of individuals recorded at each location, position on the reef, and whether the reef was exposed or sheltered is reported

**Table:**

Reef	Position	Exposure	Depth (m)	Site #	Replicate #	Morph 1	Morph 2	Morph 3	Morph 4	Total # observed
Al Fahal	Midshore	Exposed	17	1	1	2	0	0	0	2
Al Fahal	Midshore	Exposed	17	1	2	3	2	0	0	5
Al Fahal	Midshore	Exposed	17	1	3	1	0	0	0	1
Al Fahal	Midshore	Exposed	17	2	1	1	4	0	0	5
Al Fahal	Midshore	Exposed	17	2	2	3	2	0	0	5
Al Fahal	Midshore	Exposed	17	2	3	0	0	0	0	0
Al Fahal	Midshore	Sheltered	8	1	1	0	0	0	0	0
Al Fahal	Midshore	Sheltered	8	1	2	0	0	0	0	0
Al Fahal	Midshore	Sheltered	8	1	3	0	0	0	0	0
Al Fahal	Midshore	Sheltered	1	1	1	0	0	0	0	0
Al Fahal	Midshore	Sheltered	1	1	2	0	0	0	0	0
Al Fahal	Midshore	Sheltered	1	1	3	0	0	0	0	0
Al Fahal	Midshore	Sheltered	8	2	1	0	0	0	0	0
Al Fahal	Midshore	Sheltered	8	2	2	0	0	0	0	0
Al Fahal	Midshore	Sheltered	8	2	3	1	1	0	0	2
Al Fahal	Midshore	Sheltered	1	2	1	2	0	0	0	2
Al Fahal	Midshore	Sheltered	1	2	2	0	0	0	0	0
Al Fahal	Midshore	Sheltered	1	2	3	1	0	0	0	1
Fsar	Inshore	Sheltered	1	1	1	0	0	0	0	0
Fsar	Inshore	Sheltered	1	1	2	0	0	0	0	0
Fsar	Inshore	Sheltered	1	1	3	0	0	0	0	0
Fsar	Inshore	Sheltered	1	2	1	0	0	0	0	0
Fsar	Inshore	Sheltered	1	2	2	0	0	0	0	0
Fsar	Inshore	Sheltered	1	2	3	0	0	0	0	0
Fsar	Inshore	Sheltered	1	3	1	0	0	0	0	0
Fsar	Inshore	Sheltered	1	3	2	0	0	0	0	0
Fsar	Inshore	Sheltered	1	3	3	0	0	0	0	0
Shi'b Nazar	Offshore	Exposed	17	1	1	2	5	0	0	7
Shi'b Nazar	Offshore	Exposed	17	1	2	0	3	0	0	3
Shi'b Nazar	Offshore	Exposed	17	1	3	1	5	0	0	6
Shi'b Nazar	Offshore	Exposed	17	2	1	2	3	0	0	5
Shi'b Nazar	Offshore	Exposed	17	2	2	1	2	0	0	3
Shi'b Nazar	Offshore	Exposed	17	2	3	5	5	0	0	10
Shi'b Nazar	Offshore	Sheltered	1	1	1	0	0	0	0	0
Shi'b Nazar	Offshore	Sheltered	1	1	2	3	0	0	0	3
Shi'b Nazar	Offshore	Sheltered	1	1	3	0	0	0	0	0
Shi'b Nazar	Offshore	Sheltered	17	1	1	0	0	0	0	0
Shi'b Nazar	Offshore	Sheltered	17	1	2	0	0	0	0	0
Shi'b Nazar	Offshore	Sheltered	17	1	3	0	0	0	0	0
Shi'b Nazar	Offshore	Sheltered	8	1	1	0	0	0	0	0
Shi'b Nazar	Offshore	Sheltered	8	1	2	0	0	0	0	0
Shi'b Nazar	Offshore	Sheltered	8	1	3	1	0	0	0	1
Shi'b Nazar	Offshore	Sheltered	1	2	1	1	0	0	0	1
Shi'b Nazar	Offshore	Sheltered	1	2	2	0	0	0	0	0
Shi'b Nazar	Offshore	Sheltered	1	2	3	1	0	0	0	1
Shi'b Nazar	Offshore	Sheltered	17	2	1	0	0	0	0	0
Shi'b Nazar	Offshore	Sheltered	17	2	2	0	0	0	0	0
Shi'b Nazar	Offshore	Sheltered	17	2	3	0	0	0	0	0
Shi'b Nazar	Offshore	Sheltered	8	2	1	0	0	0	0	0
Shi'b Nazar	Offshore	Sheltered	8	2	2	0	0	0	0	0
Shi'b Nazar	Offshore	Sheltered	8	2	3	0	0	0	0	0
Shi'b Nazar	Offshore	Exposed	17	3	1	2	2	0	0	4
Shi'b Nazar	Offshore	Exposed	17	3	2	4	3	0	0	7
Shi'b Nazar	Offshore	Exposed	17	3	3	3	3	1	0	7
Shi'b Nazar	Offshore	Sheltered	1	3	1	1	0	0	0	1
Shi'b Nazar	Offshore	Sheltered	1	3	2	0	2	1	0	3
Shi'b Nazar	Offshore	Sheltered	1	3	3	0	0	0	0	0
Shi'b Nazar	Offshore	Sheltered	17	3	1	0	0	0	0	0
Shi'b Nazar	Offshore	Sheltered	17	3	2	0	0	0	0	0
Shi'b Nazar	Offshore	Sheltered	17	3	3	0	0	0	0	0
Shi'b Nazar	Offshore	Sheltered	8	3	1	0	1	0	0	1
Shi'b Nazar	Offshore	Sheltered	8	3	2	0	0	0	0	0
Shi'b Nazar	Offshore	Sheltered	8	3	3	0	0	0	0	0
Al Fahal	Midshore	Sheltered	1	3	1	0	0	1	0	1
Al Fahal	Midshore	Sheltered	1	3	2	0	0	0	0	0
Al Fahal	Midshore	Sheltered	1	3	3	0	0	0	0	0
Al Fahal	Midshore	Exposed	17	3	1	0	9	0	0	9
Al Fahal	Midshore	Exposed	17	3	2	0	12	0	0	12
Al Fahal	Midshore	Exposed	17	3	3	0	13	0	0	13
Abu Shosha	Inshore	Exposed	8	1	1	0	0	0	0	0
Abu Shosha	Inshore	Exposed	8	1	2	0	0	0	0	0
Abu Shosha	Inshore	Exposed	8	1	3	0	0	0	0	0
Abu Shosha	Inshore	Exposed	1	1	1	0	0	0	0	0
Abu Shosha	Inshore	Exposed	1	1	2	0	0	0	0	0
Abu Shosha	Inshore	Exposed	1	1	3	0	0	0	0	0
TahlaN	Inshore	Exposed	1	2	1	0	0	0	0	0
TahlaN	Inshore	Exposed	1	2	2	0	0	0	0	0
TahlaN	Inshore	Exposed	1	2	3	0	0	0	0	0
TahlaN	Inshore	Exposed	8	2	1	0	0	0	0	0
TahlaN	Inshore	Exposed	8	2	2	0	0	0	0	0
TahlaN	Inshore	Exposed	8	2	3	0	0	0	0	0
TahlaN	Inshore	Exposed	8	3	1	0	0	0	0	0
TahlaN	Inshore	Exposed	8	3	2	0	0	0	0	0
TahlaN	Inshore	Exposed	8	3	3	0	0	0	0	0
TahlaN	Inshore	Exposed	1	3	1	0	0	0	0	0
TahlaN	Inshore	Exposed	1	3	2	0	0	0	0	0
TahlaN	Inshore	Exposed	1	3	3	0	0	0	0	0
Fsar	Inshore	Exposed	1	4	1	0	0	0	0	0
Fsar	Inshore	Exposed	1	4	2	0	0	0	0	0
Fsar	Inshore	Exposed	1	4	3	0	0	0	0	0
Fsar	Inshore	Exposed	8	4	1	0	0	0	0	0
Fsar	Inshore	Exposed	8	4	2	0	0	0	0	0
Fsar	Inshore	Exposed	8	4	3	0	0	0	0	0
Fsar	Inshore	Exposed	1	5	1	0	0	0	0	0
Fsar	Inshore	Exposed	1	5	2	0	0	0	0	0
Fsar	Inshore	Exposed	1	5	3	0	0	0	0	0
Fsar	Inshore	Exposed	8	5	1	0	0	0	0	0
Fsar	Inshore	Exposed	8	5	2	0	0	0	0	0
Fsar	Inshore	Exposed	8	5	3	0	0	0	0	0
Fsar	Inshore	Exposed	8	6	1	0	0	0	0	0
Fsar	Inshore	Exposed	8	6	2	0	0	0	0	0
Fsar	Inshore	Exposed	8	6	3	0	0	0	0	0
Fsar	Inshore	Exposed	1	6	1	0	0	0	0	0
Fsar	Inshore	Exposed	1	6	2	0	0	0	0	0
Fsar	Inshore	Exposed	1	6	3	0	0	0	0	0
Al Fahal	Midshore	Exposed	1	1	1	3	0	0	0	3
Al Fahal	Midshore	Exposed	1	1	2	4	0	1	0	5
Al Fahal	Midshore	Exposed	1	1	3	0	0	0	0	0
Al Fahal	Midshore	Exposed	8	1	1	1	1	3	0	5
Al Fahal	Midshore	Exposed	8	1	2	2	0	0	0	2
Al Fahal	Midshore	Exposed	8	1	3	0	1	0	0	1
Al Fahal	Midshore	Exposed	1	2	1	0	0	0	0	0
Al Fahal	Midshore	Exposed	1	2	2	0	0	0	0	0
Al Fahal	Midshore	Exposed	1	2	3	0	0	0	0	0
Al Fahal	Midshore	Exposed	8	2	1	3	0	1	0	4
Al Fahal	Midshore	Exposed	8	2	2	6	1	2	0	9
Al Fahal	Midshore	Exposed	8	2	3	2	1	0	0	3
Al Fahal	Midshore	Exposed	1	3	1	0	1	0	0	1
Al Fahal	Midshore	Exposed	1	3	2	2	0	2	0	4
Al Fahal	Midshore	Exposed	1	3	3	2	2	0	0	4
Al Fahal	Midshore	Exposed	8	3	1	0	1	0	0	1
Al Fahal	Midshore	Exposed	8	3	2	3	4	1	1	9
Al Fahal	Midshore	Exposed	8	3	3	3	3	3	3	12
Al‐Dgiyg	Midshore	Exposed	8	4	1	0	0	0	0	0
Al‐Dgiyg	Midshore	Exposed	8	4	2	0	0	0	0	0
Al‐Dgiyg	Midshore	Exposed	8	4	3	0	0	0	0	0
Al‐Dgiyg	Midshore	Exposed	1	4	1	0	0	0	0	0
Al‐Dgiyg	Midshore	Exposed	1	4	2	0	0	0	0	0
Al‐Dgiyg	Midshore	Exposed	1	4	3	2	0	0	0	2
Um Al‐Balm	Midshore	Exposed	8	5	1	3	0	3	0	6
Um Al‐Balm	Midshore	Exposed	8	5	2	6	1	1	0	8
Um Al‐Balm	Midshore	Exposed	8	5	3	2	0	2	1	5
Um Al‐Balm	Midshore	Exposed	1	5	1	3	0	0	0	3
Um Al‐Balm	Midshore	Exposed	1	5	2	3	0	0	0	3
Um Al‐Balm	Midshore	Exposed	1	5	3	3	0	0	0	3
Um Al‐Kthal	Midshore	Exposed	8	6	1	3	0	0	0	3
Um Al‐Kthal	Midshore	Exposed	8	6	2	2	0	0	1	3
Um Al‐Kthal	Midshore	Exposed	8	6	3	0	1	1	0	2
Um Al‐Kthal	Midshore	Exposed	1	6	1	1	0	1	0	2
Um Al‐Kthal	Midshore	Exposed	1	6	2	1	0	2	0	3
Um Al‐Kthal	Midshore	Exposed	1	6	3	3	0	0	0	3
Shi'b Nazar	Offshore	Exposed	8	1	1	0	4	4	0	8
Shi'b Nazar	Offshore	Exposed	8	1	2	5	0	2	0	7
Shi'b Nazar	Offshore	Exposed	8	1	3	3	0	4	1	8
Shi'b Nazar	Offshore	Exposed	1	1	1	4	0	0	0	4
Shi'b Nazar	Offshore	Exposed	1	1	2	1	0	0	1	2
Shi'b Nazar	Offshore	Exposed	1	1	3	4	0	0	0	4
Shi'b Nazar	Offshore	Exposed	1	2	1	2	0	0	0	2
Shi'b Nazar	Offshore	Exposed	1	2	2	0	0	0	0	0
Shi'b Nazar	Offshore	Exposed	1	2	3	3	0	0	0	3
Shi'b Nazar	Offshore	Exposed	8	2	1	1	1	1	0	3
Shi'b Nazar	Offshore	Exposed	8	2	2	2	1	4	0	7
Shi'b Nazar	Offshore	Exposed	8	2	3	1	1	3	0	5
Shi'b Nazar	Offshore	Exposed	1	3	1	3	0	0	0	3
Shi'b Nazar	Offshore	Exposed	1	3	2	0	0	0	0	0
Shi'b Nazar	Offshore	Exposed	1	3	3	2	0	0	0	2
Shi'b Nazar	Offshore	Exposed	8	3	1	1	1	1	0	3
Shi'b Nazar	Offshore	Exposed	8	3	2	1	2	1	0	4
Shi'b Nazar	Offshore	Exposed	8	3	3	5	1	1	0	7
Abu Madafi	Offshore	Exposed	8	4	1	3	0	1	0	4
Abu Madafi	Offshore	Exposed	8	4	2	0	3	0	0	3
Abu Madafi	Offshore	Exposed	8	4	3	2	0	1	0	3
Abu Madafi	Offshore	Exposed	1	4	1	1	0	0	0	1
Abu Madafi	Offshore	Exposed	1	4	2	4	0	0	0	4
Abu Madafi	Offshore	Exposed	1	4	3	2	0	0	0	2
Abu Madafi	Offshore	Exposed	8	5	1	1	0	1	0	2
Abu Madafi	Offshore	Exposed	8	5	2	2	0	0	0	2
Abu Madafi	Offshore	Exposed	8	5	3	1	0	1	1	3
Abu Madafi	Offshore	Exposed	1	5	1	2	0	0	0	2
Abu Madafi	Offshore	Exposed	1	5	2	2	0	0	0	2
Abu Madafi	Offshore	Exposed	1	5	3	1	0	0	0	1
Abu Madafi	Offshore	Exposed	8	6	1	0	1	1	0	2
Abu Madafi	Offshore	Exposed	8	6	2	2	0	1	0	3
Abu Madafi	Offshore	Exposed	8	6	3	2	0	0	0	2
Abu Madafi	Offshore	Exposed	1	6	1	1	0	0	0	1
Abu Madafi	Offshore	Exposed	1	6	2	2	0	0	0	2
Abu Madafi	Offshore	Exposed	1	6	3	0	0	0	0	0

## APPENDIX E

### Molecular diversity indices for 1,276 bp of mitochondrial data (585 bp of COI and 691 bp of CytB, concatenated). Data are from the four color morphs as calculated in Arlequin 3.5.1.2 (Excoffier et al., 2005). Number of individuals (*N*), number of haplotypes (*N*_h_), haplotype diversity (*h*), nucleotide diversity (*π*), and Fu's *F*_S_ with associated *p* values are listed

**Table:**

	N	*N*_h_	*h*	*π*	Fu's *F*_S_
Morph 1	26	14	0.91 ± 0.04	0.002 ± 0.001	−7.74 (<0.001)
Morph 2	12	7	0.86 ± 0.09	0.001 ± 0.001	−2.92 (0.015)
Morph 3	22	12	0.92 ± 0.03	0.002 ± 0.001	−5.82 (0.001)
Morph 4	14	9	0.90 ± 0.06	0.002 ± 0.001	−3.71 (0.011)
